# Individuals with the metabolic syndrome without diabetes and/or hypertension: which risk factors should healthcare workers pay extra attention to? A longitudinal, cross-sectional study

**DOI:** 10.1136/bmjopen-2025-105379

**Published:** 2026-04-24

**Authors:** Per Nordin, Ulf Lindblad, Kristin Ottarsdottir, Bledar Daka, Margareta Hellgren

**Affiliations:** 1 Skaraborg Institute, Skövde, Sweden; 2 Family Medicine, School of Public Health and Community Medicine, Sahlgrenska Academy, University of Gothenburg Institute of Medicine, Gothenburg, Sweden

**Keywords:** Primary Care, Overweight, Prehypertension

## Abstract

**Abstract:**

**Objectives:**

To examine the metabolic syndrome (MetS) and its components as risk factors for a cardiovascular (CV) event, in individuals without diabetes and/or hypertension, and to explore which of the risk factors are the most predictive for cardiovascular disease (CVD) and whether the assessment of risk could be simplified.

**Design:**

A longitudinal, cross-sectional study.

**Setting:**

A randomly selected population.

**Participants:**

In 2002–2005, 2816 randomly selected residents of Skaraborg, Sweden, underwent physical examinations and blood tests as part of the Skaraborg Project. Exclusion of individuals with diabetes mellitus and/or hypertension at baseline left 2328 persons for analyses.

**Outcome measures:**

CV events were assessed in 2011 using national Swedish registers.

**Results:**

A total of 293 (13%) were defined as having the MetS according to the National Cholesterol Education Programme (NCEP) and 292 according to the International Diabetes Federation (IDF) definition, whereof 27 had a CV event after a mean follow-up time of 9.7 years. The MetS according to NCEP was significantly predictive of a CVD with an HR 2.4 (95% CI 1.4 to 3.9) but not according to the IDF definition. Blood pressure was significantly predictive according to both definitions (HR 1.77, 95% CI 1.06 to 2.97). Also, triglycerides (Tg) were significantly predictive for a CV event (HR 2.05, 95% CI 1.17 to 3.59). Neither waist circumference, high-density lipoprotein nor fasting plasma glucose was predictive for a CV event. Combining a blood pressure ≥125/≥80 mm Hg with Tg ≥1.5 mmol/L was predictive for CVD (HR 2.1, 95% CI 1.3 to 3.6) with a sensitivity of 32.5% and numbers needed to examine (NNE) 7.1. Lowering the cut-off for Tg to ≥1.2 mmol/L (HR 2.1, 95% CI 1.3 to 3.4) increased sensitivity to 44.9% and NNE became 8.

**Conclusions:**

Using blood pressure combined with Tg was shown to be an equally good predictor for CVD as the complete MetS in individuals without diagnosed diabetes or hypertension. Therefore, healthcare personnel should pay attention to individuals with a borderline blood pressure, and if equivalent to or equal to 125/85, continue with measuring Tg for a discussion concerning lifestyle.

**Strengths and limitations of this study**

- A relatively young population was followed with careful examinations over a 10-year period, by two specially trained nurses.
- The participation rate was high, 76%, with randomly selected participants from both a medium-sized and a small-sized city, so the study sample may be representative for other populations with a similar lifestyle.
- Usage of reliable national registers for accurate assessment of the occurrence of study outcomes.
- HbA1c was not measured in all participants at baseline, and thus, some cases of type 2 diabetes mellitus based on HbA1c criteria might have remained undiagnosed.
- The number of events was limited.

## Introduction

 The metabolic syndrome (MetS) is a relatively newly defined syndrome without a clear-cut definition and although the three most used (WHO, National Cholesterol Education Programme (NCEP) and International Diabetes Federation (IDF)) are similar,^1^ globally accepted definitions/diagnosis criteria are lacking. All definitions of the MetS include several cardiovascular (CV) risk factors, such as abdominal obesity; high blood pressure; increased triglycerides (Tg) and low levels of high-density lipoprotein (HDL) (table 1). Applying these definitions shows that the MetS is common and the syndrome is estimated to affect around 1.4 billion people worldwide with a varying degree of severity.^2^ Its presence is an indication of an increased risk to develop type 2 diabetes mellitus (T2DM)^3^ (if not already existing) and CV events.^4^ In Sweden alone, MetS in various forms is estimated to exist in around 15% of the middle-aged population.^5^

**Table 1:** Definitions of metabolic syndrome

Definition of WHO: dysglycaemia (first criteria) and ≥2 others	Definition of revised NCEP ATP III: any ≥3 of the following criteria	Definition of IDF: waist (fourth criteria) and ≥2 others
Dysglycaemia: presence of insulin resistance, or IFG, or IGT, or diagnosed T2DM.	Dysglycaemia: fasting blood glucose ≥5.6 mmol/L (100 mg/dL) or diagnosed T2DM.	Dysglycaemia: fasting blood glucose ≥5.6 mmol/L (100 mg/dL) or diagnosed T2DM.
HDL cholesterol: Men: <0.9 mmol/L (35 mg/dL); Women: <1.0 mmol/L (39 mg/dL).	HDL cholesterol: Men: <1.03 mmol/L (40 mg/dL); Women: <1.29 mmol/L (50 mg/dL) or drug treatment for low HDL-C.	HDL cholesterol: Men: <1.03 mmol/L (40 mg/dL); Women: <1.29 mmol/L (50 mg/dL) or drug treatment for low HDL-C.
Blood triglycerides: ≥1.7 mmol/L (150 mg/dL).	Blood triglycerides: ≥1.7 mmol/L (150 mg/dL) or drug treatment for elevated triglycerides.	Blood triglycerides: >1.7 mmol/L (150 mg/dL) or drug treatment for elevated triglycerides.
Waist/hip ratio: Men: >0.9; Women: >0.85 and/or BMI >30 kg/m^2^.	Waist circumference: Men: ≥102 cm; Women: ≥88 cm.	Waist circumference:* Men: ≥94 cm; Women: ≥80 cm.
Blood pressure: systolic ≥140 mm Hg and/or diastolic ≥90 mm Hg.	Blood pressure: systolic: ≥130 mm Hg; diastolic: ≥85 mm Hg or drug treatment for hypertension.	Blood pressure: systolic: ≥130 mm Hg; diastolic: ≥85 mm Hg or drug treatment for hypertension.
Other: microalbuminuria (>20 µg/min).	–	–

* For Europids, waist circumference varies between countries/ethnicities.

BMI, body mass index; HDL, high-density lipoprotein; IDF, International Diabetes Federation; IFG, impaired fasting glucose; IGT, impaired glucose tolerance; NCEP ATP III, National Cholesterol Education Programme Adult Treatment Panel III; T2DM, type 2 diabetes mellitus.

Thus, many individuals who visit primary care are affected by the MetS, some with neither established hypertension nor diabetes. These individuals are usually sent home with soothing confirmations about blood pressure and glucose status. At best, they are given advice and support for a healthy lifestyle. However, even individuals without diabetes or hypertension, affected by the MetS, are at a lifetime risk of developing cardiovascular disease (CVD) earlier than in a population not affected by MetS, particularly when affected as young.^6^ Individuals diagnosed with hypertension and/or diabetes are usually well taken care of, but what other measurements, included in the definition of MetS, are the most important to consider, in a population without diabetes and/or hypertension, is unknown.

The feasibility of using the MetS as a tool for identifying persons at risk in a clinical situation is also debatable as it requires measurements of waist circumference, blood pressure, body weight and height in addition to blood samples, not a simple task in a clinical situation characterised by lack of time. In this study, we explore which risk factors, among those used in MetS definition in a Swedish study population without diagnosed diabetes or hypertension, are most predictive for CVD and whether the assessment of risk could be simplified and thus more suitable in a clinical situation.

## Method

**Figure 1:**
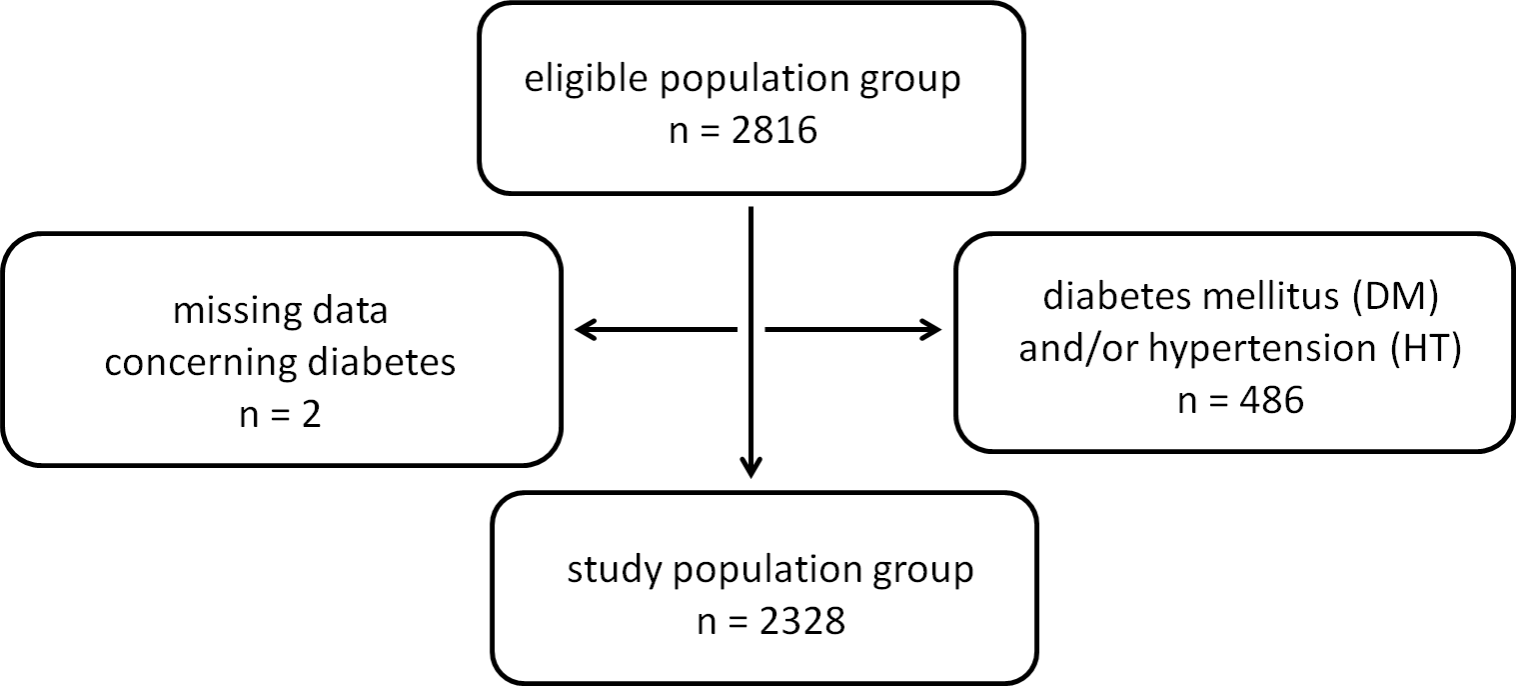
Flowchart of the study.

In the Vara-Skövde cohort within the Skaraborg project, 2816 randomly chosen individuals went through a careful health examination 2002–2005 (76% participation rate). The study population (30–75 years of age) was collected with a deliberate three-fold oversampling of individuals younger than 50 years. The protocol was designed to explore the development and progression of non-communicable diseases over time. For the present study, all individuals with baseline diagnoses of diabetes mellitus and/or hypertension were excluded (n=486), as were two individuals with missing data for diabetes status, leaving 2328 individuals for analyses (see flowchart, figure 1). Hypertension was defined according to the Blood Pressure Management JNC 7,^7^ that is, as systolic blood pressure ≥140 mm Hg and/or diastolic blood pressure ≥90 mm Hg or an adult individual using antihypertensive medication.^8^ Diagnosis for diabetes mellitus was based on the 1999 WHO recommendation^9^ or an individual previously diagnosed with diabetes.

Lifestyle factors were documented based on validated questionnaires.^10 11^ Participants were asked to estimate the number of days they had been drinking alcohol in the last month and the average amount of alcohol they had consumed during those days. An estimate of self-reported alcohol consumption (grams per week) was calculated for each participant. Based on quartiles of this estimated weekly alcohol consumption, the participants were divided into three groups. Non-drinkers were participants that reported no alcohol consumption last month. One drink of alcohol was defined as equivalent to 15 g of alcohol.^10 11^ Level of leisure time physical activity (LTPA) was self-reported in four possible levels: Sedentary LTPA, that is, mostly inactive, less strenuous LTPA <4 hours a week; medium LTPA, less strenuous LTPA >4 hours/week or high LTPA, that is, strenuous LTPA (jogging, swimming, tennis, etc) ≥2 hours/week.^12^

Smoking was classified as previous smoker, smoking, non-smoker.

### Procedures

The details of the Skaraborg Project have been described previously.^13 14^ The participants were phenotyped with body weight and height and waist and hip circumference. Auscultatory blood pressure was measured two times in the right arm after 5 min’ rest, with 1 min’s rest between measurements. Participants were in a supine position with the arm adjusted to heart level and a Tricuff was used for automatic adjustment of cuff size to arm circumference.^15^ The mean of two consecutive blood pressure readings was used for further analyses. Blood samples were drawn, and all participants performed an oral glucose tolerance test (OGTT) with ingestion of a 75 g standard glucose load after an overnight fast. Glucose measurements and cholesterol were analysed at the local hospital lab following standard procedures and other blood samples were immediately stored in a −80°C.

The CV data were collected in 2011, with a mean follow-up time of 8 years after the baseline examination and included incident non-fatal and fatal diagnoses of ischaemic heart disease (International Classification of Diseases (ICD)-10: I21, I22, I23, I25), stroke (ICD-10: I60, I61, I63, I64), percutaneous coronary intervention and coronary artery intervention by-pass grafting. These diagnoses were obtained from the National Swedish Hospital Discharge Register and the National Mortality Register.

To examine the influence of the MetS in individuals without diagnosed hypertension and/or diabetes mellitus, on the development of CVD, we chose the definition of the revised NCEP Adult Treatment Panel III (ATP III) from 2005 and the definition of IDF. We did not use the WHO definition as this definition includes only individuals with established hypertension,^16 17^ excluded in this study (table 1).

### Statistics

Descriptive statistics were reported using means, medians and proportions, SD and quartiles. To assess the various time-dependent risks for CV events, we used Cox regression models reporting HRs as primary measure. All data were adjusted for age, sex, smoking, alcohol intake and the components in the MetS. We inspected the models for violations of proportional hazards. To assess the diagnostic properties of the risk factors deemed interesting, we calculated the sensitivity, specificity, positive and negative predictive value as well as the number needed to examine. The level of significance was set at 5% and confidence level set at 95% accordingly.

### Patient and public involvement

Patients were not involved in the design of this study. However, the results will be published in a national paper addressing medical doctors overall and particularly in primary care and may have consequences for identifying patients at risk of CVD.

## Results

### Baseline characteristics

A total of 293 individuals (157 men) were defined as having the MetS using the revised NCEP ATP III definition, that is, 13% of the population without manifest diabetes or hypertension. The prevalence, using the IDF definition, was about the same, 292 individuals (162 men). Women with the MetS were older than women without the MetS, while men with and without the MetS were about the same age. Women with the MetS consumed less alcohol, but both men and women with the MetS reported more smoking than individuals without the MetS. Participants without the MetS were displaying a generally higher level of physical activity. Table 2 is based on the revised NCEP ATP III definition, but the characteristics are similar using the IDF definition (see table 2).

**Table 2:** Baseline characteristics for men and women without diabetes and/or hypertension with and without the metabolic syndrome according to NCEP ATP III

	All, n=2328		MetS, n=287		No MetS, n=2041	
	Men, n=1147	Women, n=1181	Men, n=154	Women, n=133	Men, n=993	Women, n=1048
Age, years (SD)	45 (10.3)	45 (10.2)	47 (9.9)	51 (11.2)	45 (10.3)	45 (9.8)
BMI, kg/m^2^ (SD)	27 (3.4)	26.0 (4.8)	30.0 (3.8)	30.5 (5.2)	26.0 (3.1)	25.4 (4.4)
Waist, cm (SD)	94 (9.5)	84 (12.3)	104 (9.5)	96.1 (12.4)	92.1 (8.5)	81.9 (11.4)
BP, mm Hg (SD)						
Systolic	120 (11.4)	114 (13)	126 (13.4)	126 (12.6)	119 (10.7)	113 (12.4)
Diastolic	70 (8.6)	67 (8.5)	75 (9.5)	72 (9.1)	70 (8.2)	66 (8.2)
HDL, mmol/L (SD)	1.2 (0.3)	1.4 (0.3)	0.9 (0.2)	1.07 (0.2)	1.2 (0.3)	1.5 (0.3)
LDL, mmol/L (SD)	3.4 (0.9)	3.1 (0.9)	3.6 (1.0)	3.6 (0.9)	3.3 (0.9)	3.0 (0.9)
p-TG, mmol/L (SD)	1.4 (0.9)	1.1 (0.6)	2.5 (1.2)	1.8 (0.9)	1.2 (0.7)	1.0 (0.5)
fp-glc, mmol/L (SD)	5.3 (0.5)	5.1 (0.5)	5.7 (0.5)	5.6 (0.5)	5.3 (0.4)	5.1 (0.4)
Alcohol, g/week (quartiles 25–75)	38.2 (14.0–78.3)	18.9 (3.1– 44.1)	36.8 (11.3–72.5)	12.6 (0.0– 33.4)	38.4 (14.5–80.2)	18.9 (3.8– 46.6)
Smoker, no. (%)	180 (15.7)	244 (20.7)	42 (27.3)	39 (29.3)	138 (13.9)	205 (19.6)
Physical activity						
Level 1	93 (8.1)	65 (5.5)	21 (13.6)	5 (3.8)	72 (7.3)	60 (5.7)
Level 2	565 (49.3)	734 (62.2)	93 (60.4)	99 (74.4)	472 (47.5)	635 (60.6)
Level 3	404 (35.2)	324 (27.4)	33 (21.4)	21 (15.8)	371 (37.4)	303 (28.9)
Level 4	53 (4.6)	23 (1.9)	4 (2.6)	(0)	49 (4.9)	23 (2.2)

BMI, body mass index; BP, blood pressure; fp-glc, fasting plasma glucose; g/week, grams/week; HDL, high-density lipoprotein; LDL, low-density lipoprotein; MetS, metabolic syndrome; NCEP ATP III, National Cholesterol Education Programme Adult Treatment Panel III; p-TG, plasma triglycerides.

### The risk factors in the MetS

In the selected study group, analyses of the predictive values of the different risk factors included in the MetS show that the primary risk factors, actually the only factors presenting a statistically significant risk, were blood pressure at the level indicated in the MetS, that is, ≥130/≥80 mm Hg and P-Tg ≥1.7 mmol/L (see table 3). Furthermore, P-Tg as a continuous variable was significantly predictive for CVD with an HR, by mmol/L, 1.4 (95% CI 1.1 to 1.6) as were both systolic blood pressure by mm Hg, HR 1.02 (95% CI 1.01 to 1.04) and diastolic blood pressure, HR 1.05 (95% CI 1.02 to 1.08).

**Table 3:** HR for the different risk factors included in the MetS NCEP ATP III

	HR	CI	P value
Fasting plasma glucose ≥5.6 mmol/L	1.00	0.61 to 1.68	0.138
Waist circumference ≥102 cm men; ≥88 cm women	1.08	0.61 to 1.68	0.076
Blood pressure systolic ≥130 mm Hg; diastolic ≥85 mm Hg	1.77	1.06 to 2.97	0.030
Triglycerides ≥1.7 mmol/L	2.05	1.17 to 3.59	0.012
HDL Men: <1.03 mmol/L (40 mg/dL); Women: <1.29 mmol/L (50 mg/dL)	0.72	0.40 to 1.27	0.076

HDL, high-density lipoprotein; MetS, metabolic syndrome; NCEP ATP III, National Cholesterol Education Programme Adult Treatment Panel III.

### The MetS and CVD

A total of 79 individuals were registered with a CV event over the following 10 years, of these 27 had the MetS according to revised NCEP ATP III and 22 according to the IDF definition. In our population (79 individuals without hypertension and/or diabetes), 55 of the individuals afflicted by a CV event were men, presenting an incidence of 3.4% over 8 years.

### What factors or combination of factors should be the focus of preventive measures?

The MetS NCEP was significantly predictive for CV events in a model adjusted for a range of relevant confounders, when applied on a group of individuals without manifest diabetes and/or hypertension. However, this was not the case when using the MetS IDF definition (table 4).

**Table 4:** The HR for cardiovascular disease in individuals with the metabolic syndrome without diabetes or hypertension according to the NCEP and the IDF definition, respectively

	NCEP definition			IDF definition			TgB*			TgB†		
	HR	CI	P value	HR	CI	P value	HR	CI	P value	HR	CI	P value
Crude	3.6	2.2 to 5.9	<0.001	2.5	1.5 to 4.3	<0.001	5.1	3.1 to 8.5	<0.001	5.7	3.6 to 9.0	<0.001
Model 1	2.5	1.5 to 4.1	<0.001	1.5	0.9 to 2.6	0.124	2.4	1.5 to 4.0	<0.001	2.3	1.4 to 3.8	<0.001
Model 2	2.4	1.4 to 3.9	<0.001	1.4	0.8 to 2.4	0.178	2.1	1.3 to 3.6	0.004	2.1	1.3 to 3.4	0.004

Model 2: adjusted for age, sex, smoking, alcohol intake and previous cardiovascular event.

* A combination of triglycerides ≥1.5 mmol/L and blood-pressure systolic 125 and/or diastolic 85, also adjusted for BMI.

† A combination of triglycerides ≥1.2 mmol/L and blood-pressure systolic 125 and/or diastolic 85, also adjusted for BMI.

BMI, body mass index; IDF, International Diabetes Federation; NCEP, National Cholesterol Education Programme; TgB, triglycerides and blood pressure.

A combination of Tg ≥1.7 combined with blood pressure ≥130/≥85, as used in the MetS definitions, proved to have a low sensitivity, 20%, while combining Tg ≥1.5 mmol/L with a systolic blood pressure ≥125 alternatively a diastolic blood pressure ≥85 was shown to yield a sensitivity of 32.5%, without losing much in specificity, and still, it would be enough with testing seven individuals to identify one at risk to develop a CVD. Mean survival time to event in this particular group was 7.9 (95% CI 7.6 to 8.1) years and in the population without blood pressure ≥125/85 mm Hg combined with Tg ≥1.5 mmol/L mean survival time to event was 8.0 (95% CI 7.9 to 8.0) years.

Choosing a cut-off for Tg of 1.2 mmol/L combined with a systolic blood pressure ≥125 or a diastolic blood pressure ≥85 sensitivity would improve to 45% but specificity would decrease to 89%. The MetS according to NCEP showed a sensitivity of 35%. For different combinations, see table 5.

**Table 5:** Sensitivity, specificity, positive predictive value and negative predictive value for detection of one person with risk to develop cardiovascular disease

	DM and/or HT, n=486	The MetS NCEP ATP III, n=293	Tg ≥1.7 and BP ≥130/85, n=101	Tg ≥1.5 and BP ≥130/85, n=133	Tg ≥1.5 and BP ≥125/85, n=184	Tg ≥1.2 and BP ≥125/85, n=288
Sensitivity %	58.2	35.0	20	26.5	32.5	44.9
Specificity %	85.7	88.2	96.2	95.0	92.9	88.8
PPV	45.9	29.7	19.4	24.3	28.3	12.2
NPV	90.7	90.5	96.3	95.4	94.1	97.9
Cases detected, % (n)	22.8 (111)	9.2 (27)	14.9 (15)	15.0 (20)	13.6 (25)	12.2 (35)
NNE	NR	10.5	6.4	6.4	7.1	

Different combinations of the risk factors included in the metabolc syndrome.

BP, blood pressure; DM, diabetes mellitus; HT, hypertension; MetS, metabolic syndrome; NCEP ATP, National Cholesterol Education Programme Adult Treatment Panel III; NNE, numbers needed to exam; NPV, negative predictive value; NR, not relevant; PPV, positive predictive value; Tg, triglycerides.

For comparison, we examined the predictive value of non-HDL but did not find non-HDL to be predictive for CVD in this relatively young population, not even without adjustments HR 1.1 (95% CI 0.9 to 1.4).

**Figure 2:**
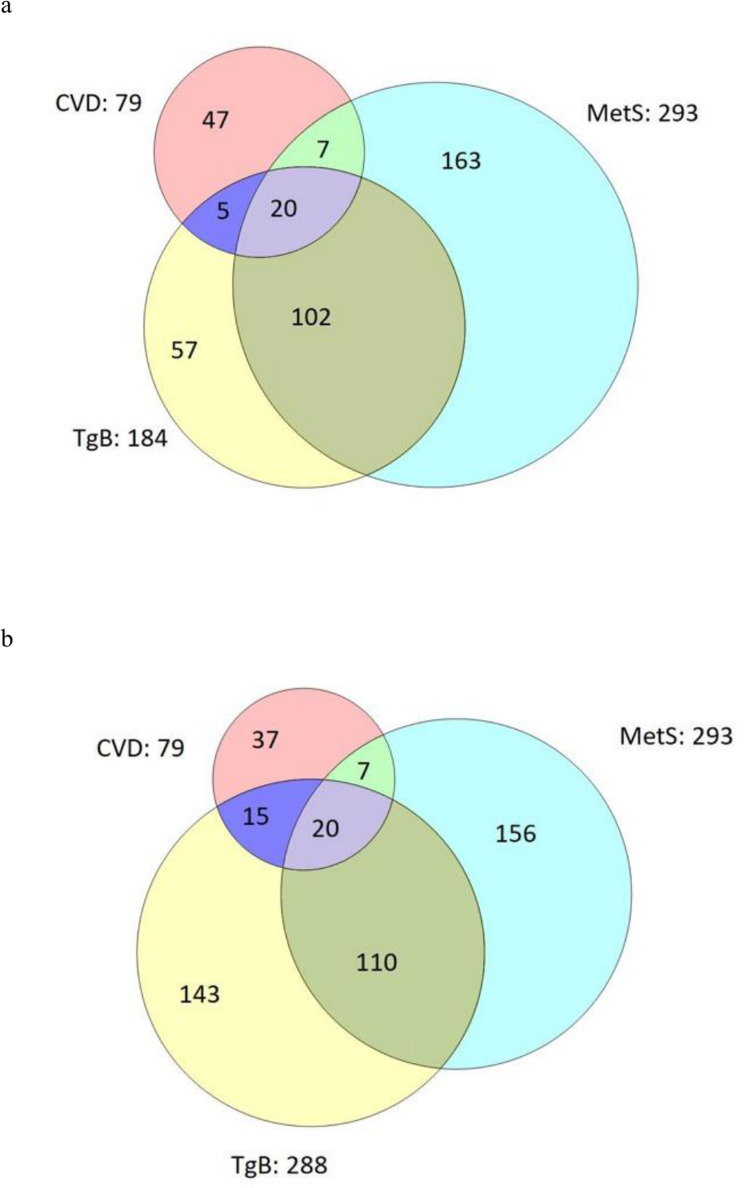
(a and b) Venn diagram illustrating the number of people at risk for a cardiovascular event predicted by the MetS according to NCEP and (a) triglycerides ≥1.5 mmol/L combined with a blood pressure ≥125/85; (b) triglycerides ≥1.2 mmol/L combined with a blood pressure ≥125/85. CVD, cardiovascular disease; MetS, metabolic syndrome; NCEP, National Cholesterol Education Programme; TgB, triglycerides and blood pressure.

When applying the MetS according to NCEP and the combinations of triglycerides and blood pressure (TgB) in a Venn diagram, we found that while 293 individuals were identified as ‘at risk’ using the MetS NCEP to find 27 individuals who developed a CVD, only 185 were defined as risk individuals using the blood pressure ≥125/85 combined with Tg ≥1.5 mmol/L finding only two people less who developed a CVD. Another option would be to use a combination of blood pressure ≥125/85 combined with Tg ≥1.2 mmol/L detecting 288 individuals at risk but also covering 35 of the persons actually developing a CVD. See Venn diagram (figure 2a,b).

We also tested for possible differences between men and women, but no interaction between sex and TgB on the development of CVD was found (p=0.387). Furthermore, as a previous CVD is associated with a considerably higher risk to develop a new CVD, the analyses were also performed excluding individuals with a previous CVD (n=43) and the main results remained statistically significant in the fully adjusted model (blood pressure, p=0.02 and Tg, p=0.012).

## Discussion

In this study, we have focused on the MetS in individuals without manifest diabetes and/or hypertension. We examined the influence of the different components of the syndrome and found that using a combination of blood pressure and plasma Tg was as good as the complete MetS according to NCEP and better than the MetS according to the IDF, to detect individuals at risk for a future CVD, in a population with the MetS but without hypertension and/or diabetes, so common in primary care.

### Strength and limitation

One strength of the study is the careful examination performed by specially trained nurses and a relatively young population followed over a substantial period. Another strength of this study is that enrolled participants were selected by random and from both a medium-sized and a small-sized city, so the study sample may be representative for other populations with a similar lifestyle. Analyses were also adjusted for several potential confounders, yielding more credibility. Other strengths include the careful examination with an OGTT and our usage of reliable national registers for accurate assessment of the occurrence of study outcomes.

This study also has some limitations. First, HbA1c was not measured in all participants at baseline, and thus, some cases of T2DM based on HbA1c criteria might have remained undiagnosed. Second, the number of events was limited and does not rule out waist circumference, HDL or blood glucose as risk factors for CVD as presented in several previous studies.^18^ Although this is likely to be expected given the age structure of the study group. Due to the limited number of events, particularly in women, an interaction with sex is difficult to rule out definitely. However, in an article from 2024, Tikhonoff *et al* examined the predictive value of different levels of Tg in 14 189 men and women and found, just as in our study, Tg to be strongly predictive for CV events, with no difference between men and women.^19^

The MetS, when including hypertension and diabetes, independent of definition, is significantly predictive for CVD and the definitions are created with the aim to identify individuals at risk. All personnel in clinical care are well aware of the risk of a CV event in patients with hypertension or diabetes, not to mention those diagnosed with both.^20^ However, the care for those patients is well organised in most healthcare settings, so the question is: what risk factors are most important for a future CV event in individuals without diabetes and/or hypertension? The MetS is still common even without including diabetes or hypertension, and in our cohort, with a mean age of 48 years, we found a prevalence of around 13%. This prevalence is also likely to increase over time.^21^ Therefore, it is of importance to establish what risk factors possibly could help to predict the development of CVD in this population.

During a visit to a healthcare facility, clinicians are likely aware of the MetS but usually focus on blood pressure, blood glucose and body weight. Most clinicians are trained to react to obesity and many healthcare units have strategies to act on individuals with borderline glucose, both impaired fasting glucose (IFG) and impaired glucose tolerance (IGT), to avoid the development of manifest T2DM. Of course, this is valuable knowledge and may induce an increased level of physical activity and a healthy diet. However, in this study, we found that waist circumference and fasting plasma glucose were not the most important predictors for CV events. It is well known that pre-diabetes (IFG and IGT) paves the way for T2DM. It is also possible to reduce the risk of developing diabetes by almost 60% for individuals with IGT, by body weight reduction and an increased level of physical activity.^22 23^ At the same time, pre-diabetes was not shown to predict CVD when adjusted for BMI, blood pressure and lipids. The risk for CVD is thus likely not due to pre-diabetes alone but more dependent on the prevalence of risk factors,^24^ even if other previous studies have reported pre-diabetes to predict CV events.^25 26^ Furthermore, the effect of a lifestyle intervention resulting in a reduced incidence of T2DM, which in turn reduces CVD over time, has only been reported in a Chinese study (the Da Qing study) after 30 years.^27 28^

Even if there are previous studies reporting waist circumference as a strong predictor of CVD and death, waist circumference did not seem to contribute to a CVD prediction model.^29^,^31^ In our cohort, including only individuals without hypertension and/or diabetes, waist circumference was not predictive for CVD when adjusted for other risk factors. Furthermore, measuring waist circumference, even if it is a simple and cheap measurement, is a time-consuming measurement. The problem with waist circumference is illustrated by how seldom it is reported in clinical care. In Sweden, waist circumference was initially included in the measurements in the national diabetes register, as being an important risk factor, but was removed as only a few of the nurses completed this particular measurement, likely due to the procedure being complex, uncomfortable and time-consuming.

Epidemiological studies have shown HDL cholesterol to have an inverse relationship to CV events, first shown in the Framingham heart study.^32 33^ The current study focuses on individuals without diabetes or hypertension, which is, no doubt, most of the population in a society, and we could not find HDL to be predictive for CVD, neither was non-HDL in this particular population. We note that independent of definition, the MetS is a good predictor for CVD in a general population, regardless of other risk factors, as stated in previous studies.^18^ However, in this study, we focus on a population without certain risk factors, and this is still a common case seen in the primary healthcare.

In this study, both blood pressure and Tg were strong predictors for future CVD, both as continuous variables and using cut-off levels as stipulated in the definitions of MetS. It is also well established that a systolic blood pressure between 130 and 140 and/or a diastolic blood pressure of 85–90 can be considered as risk factors for CVD.^34^,^36^ Furthermore, treatment resulting in a lower blood pressure level reduces the risk for a CV event. The SPRINT study could show a decreased risk for CV events by decreasing systolic blood pressure <120; however, with a certain increased risk of adverse events.^37^ The evidence was so strong that a blood pressure of more than 130/80 is now recommended for treatment in the USA.^38^ Blood pressure was also a strong predictor in our study and should be included in a simple assessment for CVD risk.

On the other hand, P-triglycerides are today, in Sweden, a forgotten or at least neglected risk factor. Discussions with colleagues in primary care show that many do not measure Tg anymore; regarding blood lipids, all focus is on low-density lipoprotein or, at best, non-HDL. Still, it is known, since many years, that Tg is a predictor for CVD in the general population.^39^ Additionally, high levels of Tg might indicate an unhealthy diet and possibly also a high alcohol intake. There are also suggestions that high levels might indicate low levels of physical activity.^40^ This means that measuring Tg could be a very relevant test related to lifestyle, to use in primary care with focus on health issues. Based on the significant risk associated with a borderline blood pressure and P-triglycerides ≥1.7, as well as based on these factors as continuous variables, it seems feasible to try to find a simpler way to estimate risk in a clinical situation rather than using the definition of MetS for this category of individuals. For such a combination of factors to work in clinical practice, the specificity needs to be high, coupled with a reasonably high sensitivity. We found that a level of ≥1.5 mmol/L seemed to be a good compromise if combined with blood pressure ≥125/85 to create a simple tool with high specificity, and even if sensitivity is low, it is still as good as for the traditional MetS, both for risk detection. If striving for a better sensitivity, one may choose a cut-off for Tg of 1.2 mmol/L, increasing sensitivity but consequently decreasing specificity. However, the only cost is the time spent on lifestyle counselling. Time needed to treat is a very relevant issue today.^41^ One way to save time is to use only blood pressure and Tg and save the time, around 10 min per patient, that it takes to measure waist circumference—an easy and cost-effective way to estimate risk. This study indicates that risk prediction can be made in a very simple way in primary care by first measuring blood pressure and if systolic blood pressure ≥125 and/or diastolic blood pressure ≥85 continue with a blood sample measuring Tg. Which cut-off to choose 1.2 mmol/L or 1.5 mmol/L may be a subject to further discussions.

### Future research

It is necessary that our findings are validated in future research, comprising larger datasets including both men and women, providing a greater number of CV events in the defined population at risk.

### Conclusions

Using blood pressure combined with plasma Tg was shown to be an equally good predictor for CVD as the complete MetS in individuals without diagnosed diabetes or hypertension. Therefore, healthcare personnel should pay attention to individuals with a borderline blood pressure and, if equivalent to 125/85, continue with measuring plasma Tg for a discussion concerning lifestyle.

## Data Availability

Data are available upon reasonable request.
