# Supplementary material for: Individuals with the metabolic syndrome without diabetes and/or hypertension: which risk factors should healthcare workers pay extra attention to? A longitudinal, cross-sectional study
Source: BMJ Open. 2026 Apr 24;16(4):e105379. doi: 10.1136/bmjopen-2025-105379 (PMC13110558; doi:10.1136/bmjopen-2025-105379)
Supplement: Supplementary file 1 [file bmjopen-2025-105379.reviewer_comments.pdf]

PEER REVIEW HISTORY

BMJ Open publishes all reviews undertaken for accepted manuscripts. Reviewers are asked to complete a checklist review form and are provided with free text boxes to elaborate on their assessment. These free text comments are reproduced below.

ARTICLE DETAILS

Title (Provisional)

Individuals with the metabolic syndrome without diabetes and/or hypertension – Which risk factors should health care workers pay extra attention to? A longitudinal, cross-sectional study

Authors

Nordin, Per; Lindblad, Ulf; Ottarsdottir, Kristin; Daka, Bledar; Hellgren, Margareta

---

VERSION 1 - REVIEW

Reviewer 1

Name Feng, Bing

Affiliation Pennington Biomedical Research Center, Louisiana State University System

Date 17-Sep-2025

COI None

In this manuscript, Nordin et al observed 2,328 Swedish adults without diabetes or hypertension for nearly a decade to study how parts of metabolic syndrome (MetS) relate to heart disease risk. They found that MetS defined by NECP, but not IDF, was linked to future cardiovascular events, with higher blood pressure and triglycerides standing out as the key predictors. In fact, just checking for borderline blood pressure along with raised triglycerides flagged risk about as well as the full MetS criteria. The following minor changes would be really appreciated.

1. In procedures, the description of blood pressure measurements appears inconsistent: initially, two readings are mentioned, but later the mean of three readings is reported. Please clarify which protocol was followed.

2. This manuscript reports a mean follow-up of 8 years. It would be helpful to clarify whether follow-up data were complete for all participants and to describe how any potential loss to follow-up was addressed.

3. The authors recognize the limited number of events among women; however, the discussion could further elaborate on the implications, such as whether future research should increase female representation or specifically examine sex-related risk factors.

4. It is recommended to add a part in which the authors can summarize all the progress of current research results in this field and put forward one's views on future research in this direction and point out the guide for follow-up researchers in the conclusion.

Reviewer **2**

Name **Xu, Shaoyong**

Affiliation **Xijing Hospital, Fourth Military Medical University, Department of Endocrinology**

Date **03-Feb-2026**

COI **None**

This study focuses on a clinically relevant yet underrecognized population - individuals with metabolic syndrome (MetS) but without diabetes and/or hypertension, and explores predictive factors for cardiovascular disease (CVD) risk, aiming to inform simplified risk screening in primary care. However, several issues require attention:

1. The study aims to explore the MetS-CVD association in individuals without diabetes/hypertension. Baseline exclusion only ruled out diabetes/hypertension, not pre-existing CVD. Though "previous CVD" was included as a confounder, the Cox regression used to analyze time-dependent follow-up risk inherently focuses on newly occurring CVD. Including pre-existing CVD violates the model’s "risk set homogeneity" assumption, introduces reverse causation bias (e.g., CVD may induce MetS-related abnormalities), and confounds the intended association, rendering HR estimates unreliable for reflecting MetS’s true predictive value for CVD in this population.

Additionally, mixing pre-existing CVD patients with the target population (diabetes/hypertension-free) may distort MetS prevalence: since MetS is a known CVD risk factor, pre-existing CVD patients often have higher MetS prevalence, leading to potential overestimation of MetS prevalence in the intended study population.

2. During follow-up, only 79 CVD events were reported (27 in the NCEP-defined MetS group, 22 in the IDF-defined MetS group), corresponding to an overall incidence rate of 3.4% over 8 years. This limited number of events raises substantial concerns regarding statistical power:

Negative findings for certain MetS components (e.g., waist circumference, HDL cholesterol, fasting plasma glucose; Table 3) may reflect insufficient power to detect true associations rather than a lack of predictive value.

Sex-specific analyses are unfeasible due to sparse events in female subgroups, and the non-significant sex interaction (p=0.387) may be a false negative.

Compounding these issues, the manuscript did not report group-level sample sizes or person-time at risk in the Cox regression models. These details are critical for interpreting hazard ratios, assessing result stability, and aligning with STROBE reporting guidelines - especially important given the low event count.

3. The study reported that the NCEP MetS definition had significant predictive efficacy for CVD, while the IDF definition did not. Additionally, waist circumference showed no significant predictive value for CVD in this diabetes/hypertension-free population (P=0.076). However, the manuscript provides no explanation for two key points: (1) why the predictive efficacy of the NCEP and IDF MetS definitions differs in this subgroup; and (2) why waist circumference lacks significant predictive value for CVD in this population.

4. The study collected physical activity data (stratified into 4 levels, Table 2) but did not adjust for it, despite physical activity being a known modifier of both MetS and CVD risk.

5. The study collected data on key covariates including smoking, alcohol consumption, and physical activity, but provided no detailed description of the data collection methods or classification criteria for these variables. This lack of transparency affects the accuracy of confounder adjustment and reduces the reproducibility of the results.

6. The rationale for selecting multiple triglyceride cutoffs (1.2, 1.5, 1.7 mmol/L) is not explained.

VERSION 1 - AUTHOR RESPONSE

Reviewer: 1
Dr. Bing Feng, Pennington Biomedical Research Center, Louisiana State University System
Thank you very much for constructive comments.

1. In procedures, the description of blood pressure measurements appears inconsistent: initially, two readings are mentioned, but later the mean of three readings is reported. Please clarify which protocol was followed.

Answer: Thank you very much for this comment. It was two readings with calculations of the mean of these readings and this is now adjusted. Thank you. Page 8

2. This manuscript reports a mean follow-up of 8 years. It would be helpful to clarify whether follow-up data were complete for all participants and to describe how any potential loss to follow-up was addressed.

Answer: Thank you for this comment. Follow-up was obtained through linkage to nationwide registers (the Swedish Cause of Death Register and the National Patient Register). The Cause of Death Register provides virtually complete ascertainment of deaths among Swedish residents, including deaths occurring abroad (which may be reported with some delay).

For hospitalisations, capture in the National Patient Register is complete for care provided in Sweden. We did not have information on emigration and therefore cannot quantify potential loss to follow-up due to individuals moving abroad; however, such migration is expected to be limited in this study population.

3. The authors recognize the limited number of events among women; however, the discussion could further elaborate on the implications, such as whether future research should increase female representation or specifically examine sex-related risk factors.

Answer: Thank you for this comment. Yes it is really a limitation since there are so few events among women. However, in a paper from 2024, Tikhonoff et al examined the predictive value of different levels of triglycerides in 14 189 men and women and found, just as in our study, triglycerides to be strongly predictive for cardiovascular events, and to be noticed with no difference between men and women (ref J Am Heart Assoc. 2024 Jan 31;13 (3):e030319). This is now added in the manuscript at page 15.

4. It is recommended to add a part in which the authors can summarize all the progress of current research results in this field and put forward one's views on future research in this direction and point out the guide for follow-up researchers in the conclusion.

Answer: Thank you for this important comment. We have actually added an extra paragraph for this particular question. Page 19

Reviewer: 2
Dr. Shaoyong Xu, Xijing Hospital, Fourth Military Medical University
Comments to the Author:
This study focuses on a clinically relevant yet underrecognized population - individuals with metabolic syndrome (MetS) but without diabetes and/or hypertension, and explores predictive factors for cardiovascular disease (CVD) risk, aiming to inform simplified risk screening in primary care. However, several issues require attention:

1. The study aims to explore the MetS-CVD association in individuals without diabetes/hypertension.
Baseline exclusion only ruled out diabetes/hypertension, not pre-existing CVD. Though "previous CVD" was included as a confounder, the Cox regression used to analyze time-dependent follow-up risk inherently focuses on newly occurring CVD.
Including pre-existing CVD violates the model's "risk set homogeneity" assumption, introduces reverse causation bias (e.g., CVD may induce MetS-related abnormalities), and confounds the intended association, rendering HR estimates unreliable for reflecting MetS's true predictive value for CVD in this population.
Additionally, mixing pre-existing CVD patients with the target population (diabetes/hypertension-free) may distort MetS prevalence: since MetS is a known CVD risk factor, pre-existing CVD patients often have higher MetS prevalence, leading to potential overestimation of MetS prevalence in the intended study population.

Answer: Thank you for addressing these important questions and giving us the opportunity to explain and clarify. Firstly, to avoid misunderstandings, the aim of this study was to explore which risk factors, among those used in MetS definition in a Swedish study population without diagnosed diabetes or hypertension, are most predictive for cardiovascular disease and whether the assessment of risk could be simplified and thus more suitable in a clinical

situation for these individuals. An important question is here raised by the reviewer – "including pre-existing CVD violates the model's "risk set homogeneity" assumption, introduces reverse causation bias (e.g., CVD may induce MetS-related abnormalities), and confounds the intended association". We will in the following develop our ideas on this. For the sake of clarity, we firstly show how the components of the MetS according to NCEP and IDF associates to CVD when we exclude previous CVD from the dataset. As can be seen, the differences are small.

Table 3. Hazard Ratio for the different risk factors included in the MetS NCEP ATPIII, adjusted for individuals with previous CVD

	HR	CI	P-value
**Fasting plasma glucose ≥5.6 mmol/L**	1.00	0.61 – 1.68	0.138
***Waist circumference ≥102 cm men ≥88 cm women**	1.08	0.61 – 1.68	0.076
**Blood pressure systolic ≥130 mmHg Diastolic ≥85 mmHg**	1.77	1.06 – 2.97	**0.030**
**Triglycerides ≥1.7 mmol/L**	2.05	1.17 – 3.59	**0.012**
**HDL Men: <1.03 mmol/L (40 mg/dl), Women: <1.29 mmol/L (50 mg/dl)**	0.72	0.40 – 1.27	0.076

Table 3. Hazard Ratio for the different risk factors included in the MetS NCEP ATPIII, individuals with previous CVD excluded

	HR	CI	P-value
**Fasting plasma glucose ≥5.6 mmol/L**	0.93	0.54 – 1.62	0.800
***Waist circumference ≥102 cm men ≥88 cm women**	1.74	1.01 – 3.01	0.047
**Blood pressure systolic ≥130 mmHg Diastolic ≥85 mmHg**	1.93	1.11– 3.34	**0.020**
**Triglycerides ≥1.7 mmol/L**	2.18	1.19 – 3.98	**0.012**
**HDL Men: <1.03 mmol/L (40 mg/dl), Women: <1.29 mmol/L (50 mg/dl)**	0.69	0.37 – 1.29	0.244

Secondly, individuals without diabetes/hypertension are a defining pre-condition in this study; however, we did not automatically exclude pre-existing CVD. It was also deemed important to use full number of CVD events after baseline to better understand the mechanism based on the defined exclusion criteria. The number of individuals with previous CVD included was 43 and among those 12 suffered another CVD event. This is the obvious reason why we adjusted for previous CVD in the regression model. To adjust for previous CVD in this situation is a well known way of dealing with such types of possible confounding and, if not applied, could lead to an overestimation of the importance of included factors in the models including the possibility of reverse causation.

We recognize the possibility or the risk that such problem, described by the reviewer, may arise. We assume that the reviewer, among other things, is talking about assumption of proportional hazards which is a pre-requisite for the Cox regression. As with every statistical model there are assumptions and it is equally true that fulfillment of these assumptions very rarely are perfectly accomplished. The assumption of proportional hazards can be expressed in a number of equivalent ways.

- *Hazard functions are proportional.*
- *Log hazard functions have a constant difference.*
- *Hazard ratios are constant over time.*
- *No substantial interactions exist between covariates and time.*

Naturally our analyses have involved checking of the assumption of proportional hazards. This can be done in several ways as outlined above. We have, among things, checked the log Hazard function and we arrived at the conclusion that the analyses are not violating this assumption in any substantial way (see figure below, model adjustments included). Furthermore, we have also conducted the same analyses on the data set where the cases of previous CVD (n=43) have been excluded and the obtained results have not deviated to any extent that would lead us to modify our interpretations. This is now added in the manuscript on page 14.

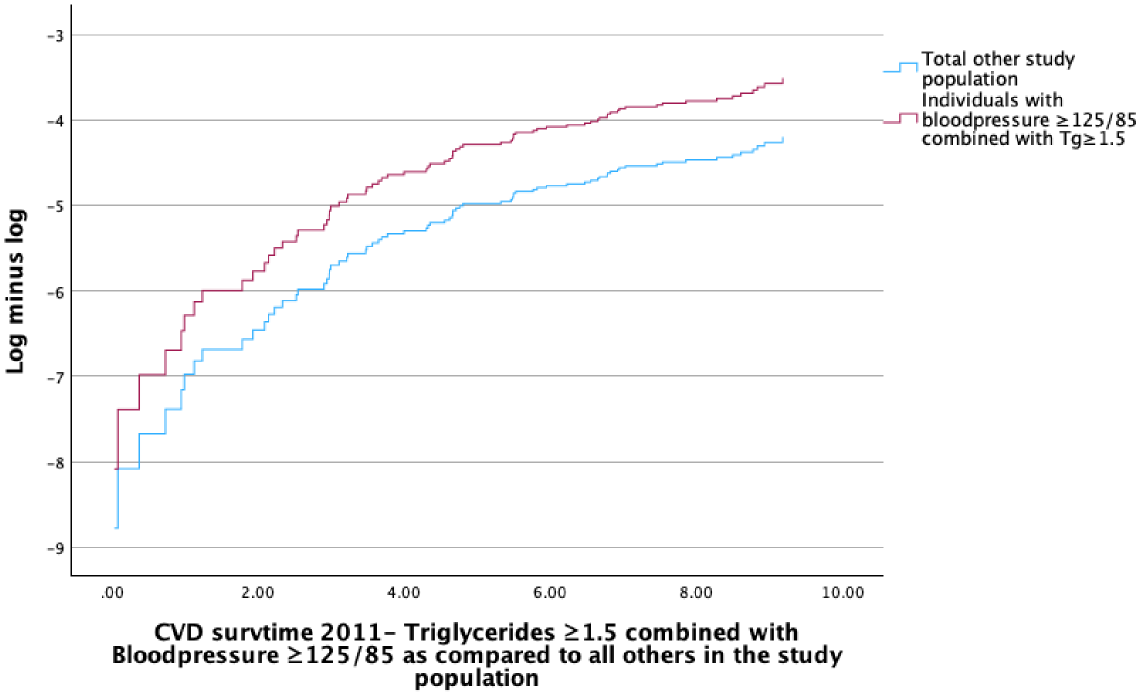

Thirdly, Concerning the prevalence of the MetS. The prevalence of the MetS is not high (13%), but rather low, in our study population (mean age 48 years) as compared to a prevalence of 22.3% in a paper by Beckett in Int J Environ Res Public Health. 2023 Sep 23;20(19):6814.

For the sake of clarity: The estimated prevalence of MetS with and without the cases of previous CVD:
With previous CVD: Number MetS cases NCEP 12.6% 294 individuals. Number MetS cases IDF 12.5% 292 individuals

Without previous CVD: Number MetS cases NCEP 12.4% 284 individuals. Number MetS cases IDF 12.3% 281 individuals

2. During follow-up, only 79 CVD events were reported (27 in the NCEP-defined MetS group, 22 in the IDF-defined MetS group), corresponding to an overall incidence rate of 3.4% over 8 years. This limited number of events raises substantial concerns regarding statistical power. Negative findings for certain MetS components (e.g., waist circumference, HDL cholesterol, fasting plasma glucose; Table 3) may reflect insufficient power to detect true associations rather than a lack of predictive value. Sex-specific analyses are unfeasible due to sparse events in female subgroups, and the non-significant sex interaction (p=0.387) may be a false negative.
Compounding these issues, the manuscript did not report group-level sample sizes or person-time at risk in the Cox regression models. These details are critical for interpreting hazard ratios, assessing result stability, and aligning with STROBE reporting guidelines - especially important given the low event count.

Answer: Thank you for this comment. True, that the number of observed endpoints is not very large and might yield a lower statistical power but this is also to be expected given the defined exclusion criterion which is at the foundation of this study. In spite of this we have found relationships that have caught our attention that might prove valuable to consider in clinical practice. Inference in statistics is obviously based on the power to detect differences and if an effect is strong it will be detected even with a lower number of information-carrying observations i.e. endpoints.
However we do know that some potentially important relationships have emerged concerning the examined population group and they could provide a simplification in clinical practice. As stated in our aim, we have focused on identifying what risk factors included in the measurements constituting the complex MetS marker that are most relevant in a population free from diabetes and hypertension with regard to CVD.
We have included further information in the manuscript concerning group level sample sizes person-time at risk in the section of limitation to elucidate further the concern raised by the reviewer on Page 13 in the manuscript.

3. The study reported that the NCEP MetS definition had significant predictive efficacy for CVD, while the IDF definition did not. Additionally, waist circumference showed no significant predictive value for CVD in this diabetes/hypertension-free population (P=0.076). However, the manuscript provides no explanation for two key points: (1) why the predictive efficacy of the NCEP and IDF MetS definitions differs in this subgroup; and (2) why waist circumference lacks significant predictive value for CVD in this population.

Answer: Thank you for these important questions which need to be clarified. Neither of the two definitions showed a particularly strong relationship with CVD events in this population group, free from diabetes and hypertension at baseline, as shown above.
The NCEP definition differs from the IDF definition concerning the measurement of waist-circumference where IDF use 94cm and 80cm for men and women respectively and NCEP 102cm and 88 cm. Based on the complex requirements for being marked as a person MetS this only corroborates our thesis that there might exist other combinations of simpler risk factor combinations that are better suited for identifying person at risk in a clinical practice.

***Key points** 1) The study population in this paper is a relatively young population and thus presenting rather few outcome events. This might increase the risk for possible differences*

between the predictive values of the two definitions. We know from previous studies that both definitions have a predictive value for CVD and in a thorough review in BMC from 2011 (BMC Med. 2011 May 5;9:48) and the authors in these papers state that multiple studies have concluded that the metabolic syndrome, independent of definition is strongly predictive for CVD. We have added a sentence about this including this reference in the paper. This paper focus on a relatively young population and have the aim to find a simpler way to assess risk in primary care in individuals free from diabetes and manifest hypertension (common in primary care and often overlooked). To assess differences in detail between different definitions of the MetS is not within the scoop of this manuscript.
2) The importance of waist-circumference. We do not question waist-circumference as a risk factor for CVD, we only state that waist-circumference is less predictive than blood-pressure and triglycerides. It is highly possible that (even likely), if we had more outcome events, waist-circumference would be presented as a risk-factor, even if it would be weaker than blood pressure and triglycerides. Blood pressure and triglycerides could be considered a part of the causal chain witch ties waist-circumference with CVD, and therefore blood-pressure and triglycerides are closer to CVD and this may explain the results. The limited amount of outcome events is presented as a limitation in the paper at page 15.

4. The study collected physical activity data (stratified into 4 levels, Table 2) but did not adjust for it, despite physical activity being a known modifier of both MetS and CVD risk.

Answer: Thank you for giving us the opportunity to clarify this question. When starting the calculations, data were tested for possible confounders and physical activity did not influence the calculations at all, and as we adjust for many important confounders we did not add adjustments for leisure time physical activity.

5. The study collected data on key covariates including smoking, alcohol consumption, and physical activity, but provided no detailed description of the data collection methods or classification criteria for these variables. This lack of transparency affects the accuracy of confounder adjustment and reduces the reproducibility of the results.

Answer: Thank you for this important comment. We have now added a detailed description concerning the collection of information about smoking, alcohol and physical activity.
Page 7-8.

6. The rationale for selecting multiple triglyceride cutoffs (1.2, 1.5, 1.7 mmol/L) is not explained.

Answer: Thank you for this comment. There is an abundance of methods available to select a proper cut-off value without any given best choice. The standard cut-off for used in all the MetS definitions for triglycerides is 1.7 mmol/L. We aimed at establishing a cut-off that, based on our clinical experience, would be able to detect enough cases at risk without accomplishing an overly large inclusion subjects. In this process we tried the aforementioned cut-off values. The guiding principles for selecting cut-off candidates were based on Receiver Operating Curves while considering sensitivity and specificity.
